# Excess Salt Increases Infarct Size Produced by Photothrombotic Distal Middle Cerebral Artery Occlusion in Spontaneously Hypertensive Rats

**DOI:** 10.1371/journal.pone.0097109

**Published:** 2014-05-09

**Authors:** Hiroshi Yao, Toru Nabika

**Affiliations:** 1 Laboratory for Neurochemistry, National Hospital Organization Hizen Psychiatric Center, Saga, Japan; 2 Department of Functional Pathology, Shimane University School of Medicine, Saga, Japan; School of Pharmacy, Texas Tech University HSC, United States of America

## Abstract

Cerebral circulation is known to be vulnerable to high salt loading. However, no study has investigated the effects of excess salt on focal ischemic brain injury. After 14 days of salt loading (0.9% saline) or water, spontaneously hypertensive rats (SHR) and normotensive Wistar-Kyoto rats (WKY) were subjected to photothrombotic middle cerebral artery occlusion (MCAO), and infarct volume was determined at 48 h after MCAO: albumin and hemoglobin contents in discrete brain regions were also determined in SHR. Salt loading did not affect blood pressure levels in SHR and WKY. After MCAO, regional cerebral blood flow (CBF), determined with two ways of laser-Doppler flowmetry (one-point measurement or manual scanning), was more steeply decreased in the salt-loaded group than in the control group. In SHR/Izm, infarct volume in the salt-loaded group was 112±27 mm^3^, which was significantly larger than 77±12 mm^3^ in the control group (p = 0.002), while the extents of blood-brain barrier disruption (brain albumin and hemoglobin levels) were not affected by excess salt. In WKY, salt loading did not significantly increase infarct size. These results show the detrimental effects of salt loading on intra-ischemic CBF and subsequent brain infarction produced by phototrhombotic MCAO in hypertensive rats.

## Introduction

Excess salt intake was associated with significantly greater risk of stroke and a non-significant trend for cardiovascular disease [1]. On the basis of the results of a meta-analysis of randomized trials, the effect of reducing dietary salt (2.0–2.3 g reduction per day) on cardiovascular events was estimated to be a significant reduction of 20% [2]. Recently, the Northern Manhattan Study demonstrated that only 12% of subjects met the AHA-recommended level of sodium consumption of less than1500 mg/day, and high sodium intake was associated with an increased risk of stroke independent of vascular risk factors [3]. In the light of physiological aspects, sodium requirement would not exceed 0.6 mmol/kg per day in mature mammals, and the daily human maintenance requirement is probably below 40 mmol (approximately 2.3 g of NaCl) per day [4]. Actually, the daily intake of sodium from Paleolithic hunter-gatherer diet, estimated to be 690 mg (approximately 1.7 g of NaCl), would have been markedly below the lowest estimate of current intake (5.8–17.3 g of NaCl) [5]. In modern industrialized society, we are consuming much more salt than biological requirements.

Cerebral circulation is vulnerable to excess salt as shown in clinical studies [1], [2]. Experimental studies have also reported detrimental effects of excess salt on cerebral arteries. A high salt diet (4% NaCl for 3 days) severely impaired both endothelium- and receptor-mediated vasodilator functions of pial arterioles of rats in the absence of detectable blood pressure changes [6]. In vitro studies showed impaired vascular relaxation mechanisms in isolated rat cerebral arteries. Middle cerebral artery (MCA) from rats fed a short-term high salt diet exhibited impaired dilation in response to acetylcholine or hypoxia [7], [8]. Excess salt induced oxidative stress, and accelerated spontaneous stroke and blood-brain barrier (BBB) disruption in stroke-prone spontaneously hypertensive rats (SHR) [9]. These experimental studies suggest that pre-existing excess salt impairs cerebral blood flow (CBF) regulation and BBB integrity, and it may finally aggravate stroke outcome. However, to the best of our knowledge, no study has investigated the relationship between excess salt and the size of subsequent brain infarction produced by middle cerebral artery occlusion (MCAO).

In the present study, we investigated the effects of excess salt on the volume of brain infarction produced by distal MCAO in the rat, and explored the hypothesis that excess salt leads to impaired CBF regulation and/or greater BBB damage in critically ischemic brain tissue.

## Materials and Methods

The National Hospital Organization Hizen Psychiatric Center Institutional Review Board approved all animal surgical and maintenance procedures.

A total of 43 male spontaneously hypertensive rats/Izumo strain (SHR/Izm) [10], [11] and 11 male Wistar-Kyoto rats/Izumo strain (WKY/Izm) were purchased from Japan SLC (Shizuoka, Japan) at the age of 8 weeks, and used at the age of 3 months. Rats were fed regular rat chow (CLEA rodent diet CE-2, containing 25% protein, Na^+^ 3.1 mg/g pellet, and K^+^ 10.2 mg/g pellet) and tap water ad libitum. Rats were randomly assigned to either the salt loading group (0.9% saline as drinking water) or the control group (tap water). The amount of drinking was determined at Day 1, Day 7, and Day 14. After 14 days of salt loading or water, rats were subjected to photothrombotic distal MCAO.

### Photothrombotic distal MCAO

Rats, which had been food-deprived overnight, were anesthetized with halothane (4% for induction, 1.5% during the surgical preparation with a face mask, 0.75% after intubation, and 0.5% for maintenance) in a mixture of 70% nitrous oxide and 30% oxygen. The right femoral artery and vein were cannulated with PE 50 tubing. The rats were endotracheally intubated with PE 240 tubing, pancuronium bromide (an initial dose of 0.3 mg followed by 0.1 mg every 30 min) was intravenously injected, and the rats were mechanically ventilated. Rats were mounted on a stereotaxic head holder in the prone position, and a burr hole 3 mm in diameter was made 1 mm rostral to the anterior junction of the zygoma and squamosal bone under an operating microscope (OPMI 111, Carl Zeiss AG, Oberkochen, Germany), revealing the distal segment of the MCA.

**Figure 1:**
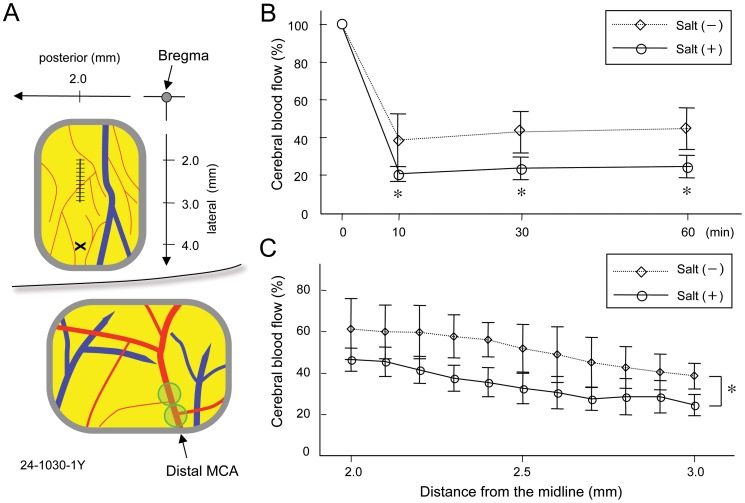
Schematic representation of cerebral blood flow (CBF) measurements and photothrombotic distal middle cerebral artery occlusion (MCAO). The laser-Doppler probe was positioned at 2 mm posterior and 4 mm lateral to the bregma (X) (A). In a subgroup of rats, regional CBF was measured at points 2 mm posterior and 2.0 to 3.0 mm lateral to the bregma by scanning the probe at 0.1 mm intervals (A). In case that it is difficult to place the “scanning” probe avoiding superficial veins and arterioles, the CBF value was substituted by the average of arbitrary units determined on neighboring two appropriate points. The lower portion of the exposed distal MCA was irradiated for 2 min with an infusion of photosensitizing dye rose bengal, and then the laser beam was moved to an additional site just proximal to the first irradiated site for 2 min (two-point hit) (A). CBF was determined at 2 mm posterior and 4 mm lateral to the bregma (B). After distal MCA occlusion, CBF was more markedly decreased in the salt-loaded group compared with the control group (n = 5–6). By means of scanning of the laser-Doppler probe, CBF was determined at 2 mm posterior and 2.0 mm to 3.0 mm lateral to the bregma (n = 3–4) (C). Values are expressed as mean±S.D. *p<0.05 vs. salt (−).

By means of photothrombosis [12], distal MCA was occluded as previously described [10], [11]. Briefly, a krypton laser operating at 568 nm (643R-Y-A01, Melles Griot Inc.) was used to irradiate the distal MCA at a power of 20 mW (an average intensity of 15.9 W/cm^2^) for 4 min (***Figure 1A***). The laser beam was focused with a 30-cm-f.l. convex lens (KPX 112, Newport Corporation, Irvine, CA, USA) and positioned onto the distal MCA after reflection from a mirror. The photosensitizing dye rose bengal (15 mg/mL in 0.9% saline; Wako Pure Chemical Industries Ltd, Osaka, Japan) was administered intravenously to a body dose of 20 mg/kg over 90 sec starting simultaneously with 4 min of laser irradiation.

### CBF by laser-Doppler flowmetry

Regional CBF was assessed by means of laser-Doppler flowmetry (ALF21D, Advance Co. Ltd, Tokyo, Japan) before and immediately after MCAO. The laser-Doppler probe was positioned at 2 mm posterior and 4 mm lateral to the bregma above the intact dura as previously described [11]. Laser-Doppler flowmetry is a method to estimate the blood perfusion in the microcirculation. Therefore, special attention was paid to avoid confounding effects of superfluous light, heterogeneous distribution of superficial blood vessels, and movement artifacts on laser-Doppler flowmetry [13]. For mechanical ventilation, we used a respirator with little vibration (Rodent ventilator, Model 7025, Ugo Basile, Comerio VA, Italy), which does not affect the laser-Doppler flowmetry by movement artifacts even placed on the experimental table.

In a subgroup of rats, regional CBF was assessed by laser-Doppler flowmetry at points 2 mm posterior and 2.0 to 3.0 mm lateral to the bregma by scanning the laser-Doppler probe at 0.1 mm intervals with a stereotaxic device (Type SR-5N and SM-11, Narishige Scientific Instrument Laboratory, Tokyo, Japan) before and 30 min after distal MCAO (***Figure 1A***).

### Infarct volume

Forty-eight h after distal MCAO, rats were decapitated under deep anesthesia, and brains were rapidly removed. The brain was cooled in ice-cold saline for 3 min and was cut into 2 mm-thick coronal sections in a cutting block. Then the slices were stained with 1% 2,3,5-triphenyltetrazolium chloride (Wako Pure Chemical Industries Ltd., Osaka, Japan) at 37°C for 30 min in the dark. The infarct area was measured in each slice with NIH Image software (ImageJ 1.46r). The infarct volume was calculated by numerical integration according to the trapezoidal rule: V = d[1/2(Y_1_+Y_n_)+Y_2_+…+Y_i_+…+Y_n−1_], where V indicates volume; d, distance between the sections; and Y_i_, cross-sectional area of ith section, and where the ends (Y_1_ and Y_n_) are equal to zero.

### Brain albumin and hemoglobin assays

Extravasation of Evans Blue has been used for assessing disruption of BBB after MCAO in animal models owing to its useful property of binding to serum albumin [14]. Albumin and hemoglobin contents determined with surface enhanced laser desorption/ionization-time of flight-mass spectrometry (SELDI-TOF-MS) in ischemic brain tissue were increased at 48 h after MCAO in our SHR stroke model (unpublished observation). Therefore, in this study, we attempted to directly measure brain albumin and hemoglobin contents as indices of BBB disruption using SELDI-TOF-MS [15], [16]. At 48 h after MCAO, the brain was sampled after transcardial perfusion with 50 mL of saline under deep anesthesia. In the present study, we defined ischemic penumbra as the region that is at risk of being recruited into infarction according to the classic concept of ischemic penumbra (i.e., the condition of an ischemic brain with CBF between the upper threshold of electrical silence and the lower threshold of energy and ion pump failure *at early stages of* focal ischemia) [10]. The sampling procedure of discrete brain regions (A and B: ischemic core, C: penumbra, and D: contralateral side), and the methods of SELDI-TOF-MS were previously described [17].

The brain sample was sonicated in 100 µL of ice-cold Buffer 1 (50 mM Tris HCl, 150 mM NaCl, 0.5% Triton X100, pH 7.4). After centrifugation (14,000X g, 4°C, 20 min), supernatant was used for albumin and hemoglobin assays by using weak cation-exchange (CM10) ProteinChip Arrays (BIO-RAD Laboratories Inc., Hercules, CA, USA). Prior to sample loading, CM10 arrays were equilibrated with 5 µL of binding/washing buffer (50 mM Gly-HCl, pH 3). Protein content was determined by the Bradford method, and 2 µL of sample (5.0 µg protein equivalent) was applied to the array. The array was washed 3 times with 5 µL of binding/washing buffer, followed by a brief water wash. After air-dried, a 0.5 µL aliquot of saturated sinapinic acid solution (an energy absorbing molecule) dissolved in 50% acetonitrile containing 0.5% trifluoroacetic acid was added twice and allowed to dry. The ProteinChip Array was then transferred into the ProteinChip Biomarker System (BIO-RAD Laboratories Inc., Hercules, CA, USA), which generates nanosecond laser pulses from a UV-emitting pulsed nitrogen laser. With the time-of-flight mass spectrometry, the mass-to-charge (m/z) or molecular weight (M.W.) of the peptide or protein was determined. The m/z of each of the protein was determined relative to external calibration standards: bovine insulin (5,733.6 Da), bovine cytochrome C (12,230.9 Da), bovine β lactoglobulin A (18,363.3 Da), and horseradish peroxidase (43,240.0 Da).

### CBF autoregulation

Bilateral femoral arteries were cannulated with PE 50 tubing: one arterial catheter was used for continuous blood pressure monitoring, and the other for withdrawal of blood to induce controlled hemorrhagic hypotension. Regional CBF was continuously monitored at 2 mm posterior and 4 mm lateral to the bregma. After 30 min of stabilization, baseline values of CBF were assessed at least three times, and arterial blood was withdrawn from the femoral arterial catheter to decrease systemic blood pressure in a stepwise manner by 10 mmHg/step. Indices for lower limits of CBF autoregulation (CBF decreased by 10% or 20% from the baseline, and MABP ranges from baseline to lower limits) were compared between the salt-loaded group and the control group as previously described [18].

### Normotensive Wistar-Kyoto rats

After 14 days of salt loading or water, 11 WKY/Izm were subjected to photothrombotic distal MCAO. Forty-eight h after distal MCAO, rats were decapitated under deep anesthesia, and infarct volume was determined.

### Statistical analysis

Data are expressed as mean±S.D. Intergroup comparisons were made with the unpaired two-tailed t-test. Intraischemic CBF values were compared with analysis of variance followed by Bonferroni post hoc test. The amounts of drinking over time in the salt-loaded and the control groups were analyzed by two-way analysis of variance with Bonferroni post hoc test to correct for multiple comparisons. A significance level of 0.05 was used in all analyses. The data were analyzed with IBM SPSS 18.0 for Windows.

## Results

### Salt preference in SHR

**Figure 2:**
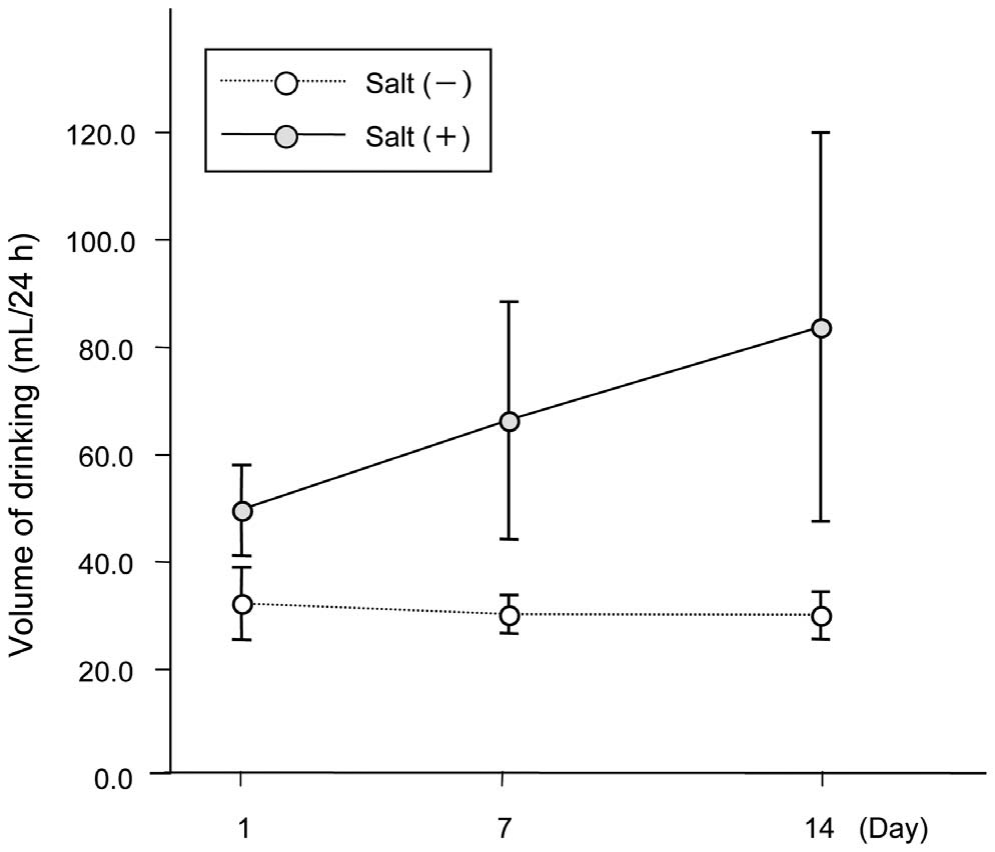
Amount of drinking of water or saline (0.9% NaCl) determined at Day 1, Day 7, and Day 14. The volume of water intake was stable during the experimental period, while the volume of saline intake progressively increased from Day 1 to Day 14. Values are mean±S.D. (n = 8–9).

The volumes of drinking water during 24-hour period were approximately 30 mL in the control group, while SHR showed an exaggerated preference for NaCl (n = 8–9) (***Figure 2***). Two-way analysis of variance revealed a significant effect of saline on the volumes of drinking (p<0.001), and a strong tendency to an increased intake of saline from Day 1 to Day 14 (p = 0.052).

### Physiological variables

Baseline physiological variables in SHR/Izm are presented in ***Table 1***. Head and rectal temperatures, blood gases, and glucose were maintained within the normal range, and were not different between the groups. Resting MABP was 137±15 mmHg in the salt loaded group, which was almost the same with 141±7 mmHg in the control groups. Changes in MABP after MCAO were also not different between the groups (data not shown). There was no difference in plasma sodium concentration between the groups, while plasma potassium concentration tended to be reduced in the salt-loaded group compared with the control group (p = 0.078).

**Table 1:** Physiological variables.

		salt (−)	salt (+)
		n = 19	n = 24
Body weight	(g)	298±14	303±18
Mean arterial blood pressure	(mmHg)	141±7	137±15
Head temperature	(°C)	36.4±0.2	36.4±0.2
Rectal temperatue	(°C)	37.2±0.3	37.2±0.3
Blood gases			
pCO_2_	(mmHg)	39.9±2.7	39.7±3.4
pO_2_	(mmHg)	111±9	110±10
pH		7.41±0.02	7.42±0.02
Fasting blood glucose	(mmol/L)	7.37±0.98	7.48±0.80
Electrolytes			
Sodium	(mmol/L)	141±1.9	140.6±1.2
Potassium	(mmol/L)	3.82±0.21	3.68±0.27

Values are mean±S.D.

Baseline physiological variables were not different between the groups (see details in the text).

### CBF and infarct volume

After distal MCAO, CBF decreased more markedly to 20±4% of the pre-occlusion value at 10 min, 23±6% at 30 min, and 24±6% at 60 min in the salt-loaded group compared with 38±14% at 10 min, 42±11% at 30 min, and 44±11% at 60 min in the control group (p<0.05, analysis of variance followed by Bonferroni test) (***Figure 1B***).

The scanning method failed in 2 rats because of unavoidable superficial blood vessels. The scanned CBF assessed 30 min after MCAO decreased to 61±15% of the pre-occlusion value and 38±6% at 2 and 3 mm lateral, respectively, in the control group, and 46±6% and 24±5% at 2 and 3 mm lateral, respectively, in the salt-loaded group (***Figure 1C***). The CBF value, expressed as an area under curve, was significantly decreased in the salt-loaded group compared with that in the control group (34±6 vs. 51±11, p = 0.045).

**Figure 3:**
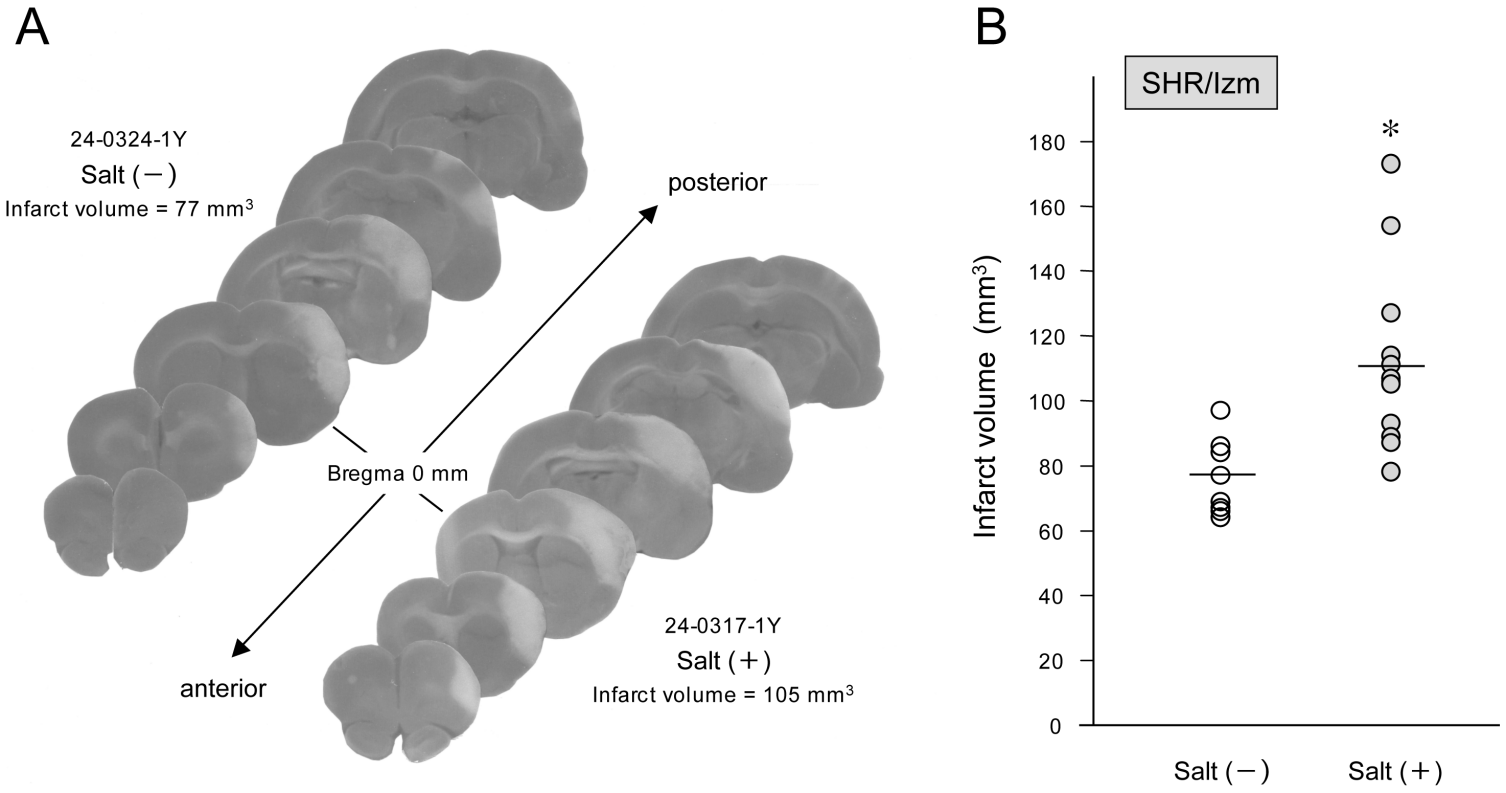
Infarct volume after distal middle cerebral artery occlusion (MCAO) in the salt-loaded and the control groups in SHR/Izm. Representative brain sections stained with 2,3,5-triphenyltetrazolium chloride from spontaneously hypertensive rats/Izumo strain (SHR/Izm) subjected to distal middle cerebral artery occlusion (MCAO) 48 h earlier (A). Average-sized cortical infarcts in rats with or without salt loading are presented. Infarct volume after distal MCAO in the salt-loaded group was significantly increased compared to the control group (*p = 0.002, n = 9–13) (B). Bars represent mean values.

Two salt-loaded and 1 control SHR/Izm died during the 48 h of survival period after MCAO. Infarct volume in the salt-loaded group was 112±27 mm^3^, which was significantly larger than 77±12 mm^3^ in the control group (p = 0.002) (***Figure 3B***).

### BBB breakdown after MCAO

**Figure 4:**
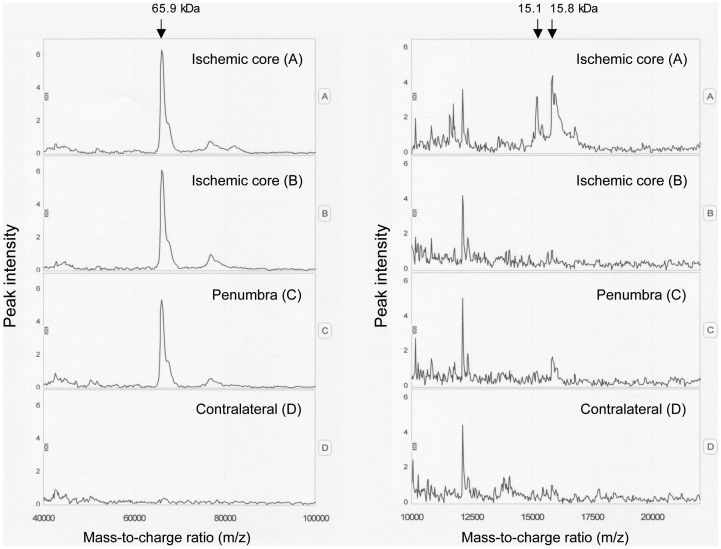
Representative spectra of albumin (left) and hemoglobin (right) in the ischemic core (A and B), penumbra (C), and contralateral zone (D). The spectrum was plotted by peak intensity against mass-to-charge ratio (m/z). The peaks corresponding to albumin (65.9 kDa, left) and hemoglobin α and β (15.1 and 15.8 kDa, respectively, right) are marked with arrows. At 48 h after middle cerebral artery occlusion (MCAO), brain albumin contents were increased by approximately 12- to 20-fold in both the ischemic core and penumbra compared with the contralateral side, while hemoglobin peaks were increased slightly only in the ischemic core.

Examples of the protein/peptide profile of samples from ischemic core, penumbra, and contralateral side are shown in ***Figure 4***. Albumin peaks were detected at m/z = 65.9 kDa, and the contents were expressed as peak intensities. Hemoglobin peaks, identified at m/z = 15.1 kDa (hemoglobin α) and at m/z = 15.8 kDa (hemoglobin β), were increased slightly only in the ischemic core. At 48 h after MCAO, brain albumin contents were increased by approximately 12- to 20-fold in both the ischemic core and penumbra compared with the contralateral side. Albumin and hemoglobin levels in discrete brain regions were not different between the salt-loaded and the control groups (***Table 2***).

**Table 2:** Albumin and hemoglobin levels in discrete brain regions.

		Albumin		Hemoglobin α		Hemoglobin β	
		Salt (+)	Salt (−)	Salt (+)	Salt (−)	Salt (+)	Salt (−)
A	Ischemic core	6.06±0.65	6.24±0.25	2.92±0.95	2.64±0.56	3.26±1.05	3.16±0.83
B	Ischemic core	5.63±0.68	5.62±1.04	ND	ND	1.53±0.39	1.08±0.33
C	Penumbra	4.99±1.17	5.62±0.20	0.48±0.30	0.95±1.05	1.37±0.32	1.94±1.44
D	Contralateral	0.41±0.18	0.32±0.10	ND	ND	ND	ND

Values are peak intensities after total current normalization (mean±S.D., n = 5). ND, not detected.

Albumin and hemoglobin levels were not different between the salt-loaded and the control groups (see details in the text).

### CBF response to hemorrhagic hypotension

Baseline MABP was 144±7 mmHg and 148±3 mmHg in the salt-loaded group and the control group, respectively. CBF at each pressure level showed no difference between the groups. The lower limit of CBF autoregulation, defined as MABP at which CBF decreased by 10% of the baseline value, was 134±11 mmHg in the salt-loaded group, which was not different from 131±8 mmHg in the control group: more detailed data are presented in ***Table 3***.

**Table 3:** Lower limits of CBF autoregulation.

		salt (−)	salt (+)
Baseline MABP	(mmHg)	148±3	144±7
Lower limits of autoregulation			
CBF decreased by 10%	(mmHg)	131±8	134±11
CBF decreased by 20%	(mmHg)	111±10	121±19
Baseline to lower limits			
CBF decreased by 10%	(mmHg)	16±9	9±8
CBF decreased by 20%	(mmHg)	37±9	23±17

Values are mean±S.D. (n = 5).

Abbreviations: MABP, mean arterial blood pressure; CBF, cerebral blood flow.

Indices for the lower limits of CBF autoregulation were not different between the salt-loaded and the control groups (see details in the text).

### Normotensive WKY

**Figure 5:**
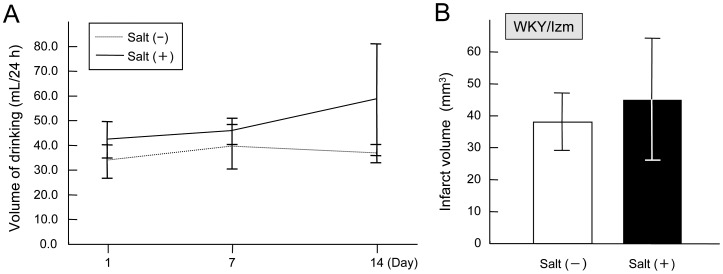
Infarct volume after distal middle cerebral artery occlusion (MCAO) in the salt-loaded and the control groups in WKY/Izm. Amount of drinking of water or saline (0.9% NaCl) determined at Day 1, Day 7, and Day 14 in Wistar-Kyoto rats/Izumo strain (WKY/Izm)(A). Infarct volume after distal middle cerebral artery occlusion (MCAO) was not significantly different between the salt-loaded and the control groups. Dada are presented as mean±S.D. (n = 5–6) (B).

Two-way analysis of variance revealed a significant effect of saline on the volumes of drinking (p = 0.004), but not significant for Day 1, 7 and 14 (p = 0.159) (***Figure 5A***). Resting MABP was 98±16 mmHg in the salt loaded group, which was not significantly different from 100±14 mmHg in the control groups. Body weight, head and rectal temperatures, blood gases, and glucose were within the normal range, and were not different between the groups (data not shown). Infarct volumes were not different between the salt-loaded and control groups (45±19 mm^3^ vs. 38±9 mm^3^, respectively) (***Figure 5B***).

## Discussion

This is the first study to demonstrate that intake of salt per se, distinct from high blood pressure, exerts detrimental effects on intra-ischemic CBF and subsequent brain infarction produced by photothrombotic distal MCAO. The extents of BBB disruption and hemorrhagic transformation, detected as increased albumin and hemoglobin contents in the ischemic brain tissue, were not different between the salt-loaded and the control groups. Because CBF response to hypotension without MCAO was not different between the salt-loaded and control groups, the extent of CBF reduction was considered to be the same provided that the reductions in local perfusion pressure in the ischemic zone were the same with or without excess salt. In this context, more markedly decreased CBF after MCAO in the salt-loaded group than in the control group may indicate that the local perfusion pressure after MCAO was compromised by salt loading, probably due to excess salt-induced dysfunction of collateral arteries (i.e., arteriole-to-arteriole anastomosis or leptomeningeal anastomosis) [19].

Laser-Doppler flowmetry is an easy method to monitor a relative measure of blood perfusion, and is commonly used in small animals. We determined CBF with laser-Doppler flowmetry “scanning” method in addition to one point measurement: a laser-Doppler flowmetry probe was laterally scanned, and CBF of the distal MCA territory was measured at points 2 mm posterior and 2.0 to 3.0 mm lateral to the bregma by scanning the laser-Doppler probe at 0.1 mm intervals, and 11 CBF values in one rat were transformed to an area under curve according to the trapezoidal rule. This “scanning” method worked well in 7 of 9 rats (78%), but drawbacks in this manual technique was that the method was time-consuming and required much effort. Nevertheless, we could show excess salt-induced more profound CBF reduction by use of two ways of laser-Doppler flowmetry in collaterally perfused ischemic regions.

In cerebral arteries, acetylcholine induces vasodilatation through a nitric oxide (NO)-mediated process, although there is also evidence suggesting a role for endothelium-derived hyperpolarizing factor in vasodilatation of cerebral arteries. A short-term elevation of dietary salt intake impaired the dilator function of in situ cerebral microcirculation without blood pressure changes [9]. In vitro studies showed that the impaired dilatation in response to acetylcholine in cerebral arteries of rats on a high-salt diet was due to attenuated NO release, while vascular response to bradykinin and to NO donor, and levels of reactive oxygen species were unaltered by elevated dietary salt intake [7], [8]. Although even a short-term high salt diet decreased the ability of blood vessels to relax, low dose angiotensin II infusion restored normal vascular function following short-term (3 days) intake of high salt diet (4.0% NaCl). Hence, salt-induced angiotensin II suppression caused a profound impairment of vascular relaxation [20]. Taken together, increased infarct volume observed in the present study could occur with excess salt if this high salt leads to impaired vascular relaxation of collateral arterioles or leptomeningeal anastomoses after distal MCAO.

The earliest phase of ischemic injury characterized by cytotoxic edema (swelling due to osmotically active molecules such as sodium from the extracellular to intracellular space) - closely coupled with ionic edema (transport of molecules such as sodium across the BBB to replace the molecules depleted by cytotoxic edema) - is followed by the second phase of the breakdown of the BBB with leakage of plasma proteins such as albumin into brain extracellular space (i.e., vasogenic edema) [21]. Salt loading increased superoxide production in the brain of SHRSP, and angiotensin II infusion in salt-loaded SHRSP significantly impaired BBB, which was prevented by an angiotensin II type 1 receptor blocker candesartan but not by a calcium channel blocker amlodipine, and this effect was independent of blood pressure level [9]. In contrast to spontaneous stroke, a decrease in apparent diffusion coefficient (ADC) of water (i.e., cytotoxic edema) was detected after 2 h of MCAO, but the effects of excess salt on ADC after MCAO were not examined in the study by Guerrini et al. [22]. Subsequent to cytotoxic and ionic edema characterized by increased sodium contents in ischemic tissue [21], [23], vasogenic edema occurs as also shown in the present study. In the present study, however, excess salt did not affect the extent of brain albumin levels in ischemic brain regions in the salt-loaded group compared with the control group.

In acute cerebral ischemia, up-regulated matrix metalloproteinases, especially gelatinases (matrix metalloproteinases-2 and 9), are closely associated with BBB disruption, edema formation, and hemorrhagic transformation [24], [25]. Yamashita et al. demonstrated that tPA administered just before the reperfusion of 4.5 h suture MCAO, induced dissociation of neurovascular unit (i.e., the detachment of astrocyte endfeet from the basement membrane), which was prevented by a free radical scavenger, edaravone [26]. Henning et al. demonstrated an unexpectedly high incidence of parenchymal hematomas at later time points (between days 1 and 4 after 30 min of suture occlusion), using gradient-echo magnetic resonance imaging [27]. In the present study, however, excess salt did not increase the hemoglobin contents in ischemic brain tissue.

Although blood pressure levels were not affected by salt loading in both SHR/Izm and WKY/Izm, excess salt represented the detrimental effects on brain infarct size produced by distal MCAO in SHR/Izm. In the present study, we found that SHR/Izm did in fact exhibit an exaggerated preference for 0.9% NaCl compared with water. SHR have consistently higher preferences for NaCl than do age-matched normotensive rats [28], [29]. Nevertheless, MABP did not change after salt loading even in SHR/Izm as previously shown by radio telemetry [30]. Although WKY/Izm also showed some preference for 0.9% NaCl, salt loading caused a non-significant increase in infarct volume in normotensive WKY/Izm. Thus, excess salt aggravates brain infarction in association with hypertension.

In conclusion, we demonstrated that excess salt increased infarct size produced by photothrombotic MCAO without increasing blood pressure in SHR but not in normotensive WKY. Excess salt did not deteriorate both vasogenic edema and hemorrhagic transformation of ischemic brain tissue after MCAO. The detrimental effects of excess salt were considered to be the result of compromised CBF in the ischemic brain tissue supplied by collateral circulation. A future study will investigate the mechanisms underlying the salt sensitivity to focal brain ischemia independent of blood pressure changes. Dietary salt reduction before the onset of stroke could reduce the size of brain infarction independent of blood pressure changes in subjects with major cerebral artery occlusion.
