# Correction: Excess Salt Increases Infarct Size Produced by Photothrombotic Distal Middle Cerebral Artery Occlusion in Spontaneously Hypertensive Rats

**DOI:** 10.1371/journal.pone.0109472

**Published:** 2014-09-23

**Authors:** The PLOS ONE Staff

There is an error in affiliation 2 for author Toru Nabika. Affiliation 2 should be: Department of Functional Pathology, Shimane University School of Medicine, Izumo, Japan.
